# Development of a theory-driven implementation strategy for cancer management guidelines in sub-Saharan Africa

**DOI:** 10.1186/s43058-020-00007-7

**Published:** 2020-02-25

**Authors:** Rebecca J. DeBoer, Jerry Ndumbalo, Stephen Meena, Mamsau T. Ngoma, Nanzoke Mvungi, Sadiq Siu, Msiba Selekwa, Sarah K. Nyagabona, Rohan Luhar, Geoffrey Buckle, Tracy Kuo Lin, Lindsay Breithaupt, Stephanie Kennell-Heiling, Beatrice Mushi, Godfrey Sama Philipo, Elia J. Mmbaga, Julius Mwaiselage, Katherine Van Loon

**Affiliations:** 1 Global Cancer Program, University of California San Francisco (UCSF) Helen Diller Family Comprehensive Cancer Center, San Francisco, CA USA; 2 Ocean Road Cancer Institute, Dar es Salaam, Tanzania; 3 Muhimbili University of Health and Allied Sciences, Dar es Salaam, Tanzania

**Keywords:** Guideline implementation, Implementation strategy, Cancer guidelines, Behavior change, Intervention mapping

## Abstract

**Background:**

Despite recent international efforts to develop resource-stratified clinical practice guidelines for cancer, there has been little research to evaluate the best strategies for dissemination and implementation in low- and middle-income countries (LMICs). Guideline publication alone is insufficient. Extensive research has shown that structured, multifaceted implementation strategies that target barriers to guideline use are most likely to improve adherence; however, most of this research has been conducted in high-income countries. There is a pressing need to develop and evaluate guideline implementation strategies for cancer management in LMICs in order to address stark disparities in cancer outcomes.

**Methods:**

In preparation for the launch of Tanzania’s first National Cancer Treatment Guidelines, we developed a theory-driven implementation strategy for guideline-based practice at Ocean Road Cancer Institute (ORCI). Here, we use the Intervention Mapping framework to provide a detailed stepwise description of our process. First, we conducted a needs assessment to identify barriers and facilitators to guideline-based practice at ORCI. Second, we defined both proximal and performance objectives for our implementation strategy. Third, we used the Capability, Opportunity, Motivation and Behavior/Behavior Change Wheel (COM-B/BCW) framework to categorize the barriers and facilitators, choose behavior change techniques most likely to overcome targeted barriers and leverage facilitators, and select a feasible mode of delivery for each technique. Fourth, we organized these modes of delivery into a phased implementation strategy. Fifth, we operationalized each component of the strategy. Sixth, we identified the indicators of the process, outcome, and impact of our intervention and developed an evaluation plan to measure them using a mixed methods approach.

**Discussion:**

We developed a robust, multifaceted guideline implementation strategy derived from a prominent behavior change theory for use in Tanzania. The barriers and strategies we generated are consistent with those well established in the literature, enhancing the validity and generalizability of our process and results. Through our rigorous evaluation plan and systematic account of modifications and adaptations, we will characterize the transferability of “proven” guideline implementation strategies to LMICs. We hope that by describing our process in detail, others may endeavor to replicate it, meeting a widespread need for dedicated efforts to implement cancer guidelines in LMICs.

Contributions to the literature

- With the recent development of resource-stratified guidelines for cancer management, strategies for guideline dissemination and implementation are urgently needed.
- Research has shown that the best way to facilitate the adoption of clinical guidelines is to develop an implementation strategy designed to target barriers to guideline use.
- We provide a detailed description of our process for developing an implementation strategy at the national cancer center in Tanzania, based on a prominent behavior change theory. This process has the potential to serve as a model for others who face similar challenges of implementing new guidelines for cancer management in resource-constrained settings.

## Background

Low- and middle-income countries (LMICs) face a growing burden of cancer and a pressing need to strengthen their cancer care delivery systems. Predictions suggest that by 2030, 13 million people will die from cancer annually, and three quarters of the deaths will occur in LMICs [1]. The overall case fatality from cancer in low-income countries is approximately 75%, compared with 46% in high-income countries (HICs) [2]. This outcome gap, largely attributable to disparities in access to early detection and standard treatment, translates into millions of preventable deaths.

Effective delivery of evidence-based practice is a critical component of addressing global disparities in cancer outcomes. Evidence-based clinical practice guidelines are widely used in oncology for clinical decision-making, healthcare quality assessment, payment decisions, and training. In recent years, several international organizations have developed resource-stratified clinical practice guidelines for use in LMICs. This began with the Breast Health Global Initiative (BHGI) in 2006 [3] and was followed by the National Comprehensive Cancer Network (NCCN) in 2015 [4] and the American Society for Clinical Oncology (ASCO) in 2016 [5]. In November 2017, the African Cancer Coalition and partners announced the new NCCN Harmonized Guidelines™ for sub-Saharan Africa for prevalent cancers and supportive care categories [6].

Despite these highly publicized international efforts to develop resource-stratified clinical practice guidelines for cancer, there has been little research to evaluate the best strategies for dissemination and implementation in LMICs. Generally speaking, guideline publication alone is insufficient to result in widespread adoption into routine practice [7]. This may be especially true in LMICs, which are the target of increasing numbers of guidelines from international and national health authorities. Well-intended guidelines frequently prove ineffective in LMICs due to inadequate resources to support successful dissemination and implementation [8]. Barriers to clinical guideline implementation in general have been well described and include provider factors such as knowledge and attitude, guideline factors such as format and content, and external factors such as lack of resources, organizational constraints, heavy workload, and cultural norms [9]. Recent surveys of oncology providers in LMICs identify numerous barriers to the successful implementation of international cancer treatment guidelines, namely inadequate infrastructure and inclusion of an overwhelming amount of complex information in the guidelines [10, 11].

Considerable research from the field of dissemination and implementation science (D&IS) has shown that structured, multifaceted implementation strategies designed to target barriers to guideline use are most likely to improve guideline adherence [9, 12, 13]. Theories, models, and frameworks are increasingly used to identify the determinants of guideline use in a specific context and design interventions tailored to overcome barriers and leverage facilitators [14, 15]. Notably, the vast majority of guideline implementation research has been conducted in HICs [16]. There is growing recognition of the urgent need to investigate how to adapt proven implementation strategies to LMIC settings, as well as to develop and evaluate novel approaches for LMICs [17, 18]. In cancer care and control broadly, implementation interventions have been characterized by uneven quality and questionable impact even in HICs, likely due to the unique complexity of the field and failure of researchers to consistently embrace high-quality D&IS standards, such as adequately describing all aspects of the interventions under investigation [19]. The global oncology community has acknowledged the need for D&IS [20–22], but the development and evaluation of guideline implementation strategies for cancer management in LMICs remains an unmet need.

In preparation for the launch of Tanzania’s first-ever National Cancer Treatment Guidelines, we developed a theory-driven implementation strategy for guideline-based practice at Ocean Road Cancer Institute (ORCI) in Dar es Salaam, Tanzania. We aim to respond to calls for detailed description of how implementation interventions are developed by using the Intervention Mapping framework to describe our stepwise process in accordance with the Template for Intervention Description and Replication (TIDieR) checklist [23–25].

## Methods

### Setting

The United Republic of Tanzania is an East African country of nearly 60 million people, and Dar es Salaam is the largest city and leading commercial center. Tanzania is classified as a low-income country by the World Bank [26]. GLOBOCAN 2018 estimated 42,000 new cancer cases and over 28,000 cancer deaths per year in Tanzania [27]. In 1996, Ocean Road Cancer Institute (ORCI) was established as the national referral center for cancer in Dar es Salaam, with a mission to provide equitable, accessible, affordable, and high quality services of early detection and cancer care to the public [28]. The government of Tanzania sponsors free care to 5400 new cancer patients per year at ORCI, including radiotherapy, chemotherapy, and palliative care. Other services such as diagnostic pathology and surgery are provided at affiliated Muhimbili National Hospital and other referring hospitals and clinics throughout the country. Beginning in 2017, Tanzania’s Ministry of Health, Community Development, Gender, Elderly and Children (MoHCDGEC) commissioned development of the country’s first comprehensive National Cancer Treatment Guidelines. The process for the development of the national guidelines is described elsewhere [29]. In preparation for the planned guideline launch in 2019, a team embedded within our broader institutional collaboration between Muhimbili University of Health and Allied Sciences (MUHAS), ORCI, and the University of California, San Francisco (UCSF) (“The MUHAS-ORCI-UCSF Cancer Collaboration”) developed a dissemination and implementation strategy for ORCI using a theory-driven approach.

### Intervention Mapping

Intervention Mapping is a framework for intervention development that maps out a stepwise process from needs assessment to evaluation [30]. The six steps in Intervention Mapping integrate theory and evidence into the major program planning activities of conducting a needs and capacity assessment, developing and implementing a program, and evaluating a program’s effectiveness [24]. We used Intervention Mapping as a basis for developing a guideline dissemination and implementation strategy at ORCI in Tanzania.

#### Step 1: Needs assessment

ORCI leaders and staff previously identified a need for improved translation of evidence to practice and standardization in clinical care, which is substantiated by available data indicating that significant numbers of patients at ORCI have not received standard treatment despite resource availability [31–33]. The proposed launch of National Cancer Treatment Guidelines in Tanzania presented an opportunity to improve evidence-based practice; however, ORCI leaders recognized that a dedicated implementation effort would be necessary to ensure that the guidelines are adopted into routine practice rather than relegated to collect dust on the shelves. The goal of our needs assessment was therefore to identify what would be required in order to implement the new guidelines at ORCI. We began with brainstorming sessions [34] among members of our research team, which includes oncologists, oncology nurses, clinical and qualitative researchers, and an implementation scientist. We then held meetings with key stakeholders, including ORCI leaders, clinical managers, oncology trainees (“residents”), and patient advocates. Finally, we conducted three focus groups with ORCI oncologists, residents, radiotherapists, and nurses (Luhar et al., unpublished data, 2019). Through this formative evaluation, we identified barriers and facilitators to guideline-based practice at ORCI. We crosschecked our findings with the literature on determinants of guideline implementation.

#### Step 2: Program objectives

The main objective of our program is to develop an implementation strategy that will effectively lead to the adoption of guideline-concordant practice at ORCI. Based on the needs assessment in Step 1, we identified proximal program objectives such as expanding access to treatment guidelines, increasing familiarity with guideline content, and improving attitudes toward guideline-based practice among providers. Following the proximal objectives, we identified both behavioral and environmental performance objectives, which include increasing guideline-based decision-making and rates of guideline-concordant treatment plans made and completed, and establishing clinical systems that promote guideline-concordant practice. The long-term objectives are to reduce inappropriate variability in clinical practice and improve quality of care, patient outcomes, and resource utilization.

#### Step 3: Select theory-based methods and practical strategies

Successful implementation of clinical practice guidelines depends on uptake by care providers, which requires sustained behavior change. In order to design an intervention that would optimally target the behavior of guideline-based clinical practice, we used the Capability, Opportunity, Motivation and Behavior/Behavior Change Wheel (COM-B/BCW) framework [35]. COM-B/BCW was developed through a systematic review and synthesis of 19 existing behavior change frameworks and provides a coherent, systematic method for identifying and organizing all potential barriers to behavior change, selecting the barriers that, if modified, are most likely to lead to behavior change in a given context, and choosing evidence-based behavior change techniques most likely to be effective in overcoming targeted barriers. We categorized the key organizational-level and individual-level barriers identified in our needs assessment into the COM-B domains of Capability, Opportunity, and Motivation (Table 1). Through iterative consultation with oncology providers and clinical leaders at ORCI, we used the BCW framework to (1) select intervention functions to address each key barrier, (2) select behavior change techniques likely to help enact each intervention function, and (3) select a feasible mode of delivery for each technique (Table 2).

**Table 1:** COM-B Theoretical Domains Framework for barriers to adoption of guideline-based clinical practice at ORCI

Domain	Barriers
Physical capability	• Lack of updated context-specific clinical practice guidelines to date
	• Existing resource-stratified guidelines (e.g., NCCN Framework™) are not easily accessible
	• Limited and/or inconsistent resources may affect the ability to follow guidelines
Psychological capability	• Providers are not very familiar with existing guidelines
	• Providers are not accustomed to guideline-based practice
	• Providers do not necessarily believe that they should be following guidelines
	• More effort is required to reference guidelines than seek (or make) an experience-based decision
Physical opportunity	• Guidelines are not part of didactic education or ongoing case-based training
	• The oncologist, resident, and nurse responsible for a patient may not typically be together when a treatment plan is made or changed
	• Multiple consultants may sequentially assume responsibility for a patient during the treatment course, leading to lack of accountability in patient management
	• Inefficiencies in clinical systems impede timely completion of standard treatment
	• Poor communication and coordination among multidisciplinary providers at different institutions
Social opportunity	• Clinical norms favor decision-making based on expert opinion and individualized experiences
	• Little professional or organizational value is placed on guideline concordance
	• Nurses often do not participate in management decision-making
	• The organizational culture is hierarchical
Reflective motivation	• Predominant belief that expertise-based decisions are superior to guidelines
	• Lack of awareness of the clinical benefit of guideline-based practice
	• Perception that guidelines do not apply to local ORCI setting
	• Consultants feel that using guidelines stifles professional authority and intellect
	• Residents and nurses do not feel empowered to question guideline concordance
Automatic motivation	• Consultants take pride in expertise and expert-based decisions
	• Residents defer to consultant expertise
	• Nurses do not routinely evaluate guideline concordance of management decisions
	• Residents and nurses may fear questioning of management decisions made by consultants
	• Gap between institutional vision/mission and available resources impacts morale

**Table 2:** Behavior Change Wheel (BCW) framework for adoption of guideline-based clinical practice at ORCI

Barriers	COM-B category	Intervention functions	Behavior change techniques and mode of delivery
Guidelines not easily accessible	Physical capability	Enablement	Distribute hard copies to every unit and clinic room, soft copies to every provider (via smartphone application). Include algorithms as a reference in clinical “Diagnosis, Staging, Treatment” (DST) forms.
Lack of knowledge of guideline content	Psychological capability	Education	Teach guideline content, including evidence basis for guidelines, to providers in dedicated education session and integrate into existing curriculum for residents and nurses.
Lack of experience in guideline-based practice	Psychological capability	Training, environmental restructuring	Administer skills training in how to use guidelines and DST forms in dedicated trainings. Integrate clinical forms into a workflow that prompt providers to apply guidelines to every patient.
Providers not aware or do not believe that they should be following guidelines	Psychological capability	Education Persuasion Modeling	Publicity campaign using branding, awareness raising of regional and international efforts to develop LMIC-specific guidelines. Selected local Champions will persuade providers that they should adhere to guidelines, and model this behavior during morning conference and in clinical practice.
Consultant/resident/nurse not together when plan is made	Physical opportunity	Environmental restructuring	Team members, including residents and nurses, should round together and review guideline concordance of treatment plan, review with consultant.
Lack of accountability in patient management	Physical opportunity	Environmental restructuring	DST forms will be completed for every patient, with documentation of rationale for treatment decisions. One consultant should be assigned to each patient at intake and ultimately responsible for treatment plan.
Current norm is that consultants make decisions based on expertise	Social opportunity	Training Modeling	Champions will model guideline-based practice on an ongoing basis on rounds/in conference. All providers (consultants, residents, nurses) will be trained to discuss or question the guideline concordance of their patients’ treatment plans. Champions will also model this behavior.
Little professional value placed on guideline concordance	Social opportunity	Incentivization	Champions will provide recognition and praise for guideline-concordant management. Planned outcomes evaluation will include audit and feedback.
Belief that expertise-based decisions are better than guidelines	Reflective motivation	Training Persuasion	Train providers in the benefits of guideline-based practice and provide evidence that they should be used in favor of expert opinion.

#### Step 4: Program plan

We organized the behavior change techniques and modes of delivery derived in Step 3 into a phased implementation strategy, summarized in Table 3. The focus of phase 1 is guideline dissemination, with distribution of hard and soft copies and a publicity campaign. Phase 2 includes dedicated knowledge and skills training at a National Cancer Treatment Guideline Summit, and phase 3 encompasses ongoing reinforcement through clinical systems restructuring, point-of-care clinical forms, and behavior modeling and promotion of guideline adherence by Implementation Champions (“Champions”).

**Table 3:** Summary of phased implementation strategy derived from the BCW/COM-B framework

Summary of phased implementation strategy	
Phase 1: Guideline launch	
• Guideline distribution: hard and soft copies (via AgileMD, Inc. smartphone application)	
• Publicity campaign for guideline implementation effort “brand” with announcements, flyers	
• Awareness raising of regional and international efforts to implement context-specific guidelines	
Phase 2: National Summit for Guideline Training	
• Dedicated teaching about benefits of guideline-based practice and regional efforts (knowledge)	
• Dedicated teaching of guideline content including evidence basis for guidelines (knowledge)	
• Dedicated training in guideline-based practice (skills)	
• Dedicated training in DST form completion (skills)	
• Dedicated workshop focusing on monitoring and evaluation of implementation strategy, including outcome measurement	
• Separate dedicated training for Champions	
Phase 3: Ongoing reinforcement of guideline-based practice	
• Champions will:	
○ Model guideline-concordant practice on an ongoing basis on rounds and during institutional conferences	
○ Routinely discuss guidelines (or supporting evidence basis) basis during rounds and conferences and encourage other providers to do so, including residents and nurses	
○ Provide academic recognition for actions to promote guideline-concordant care	
• Documentation: DST form completion, inclusion of rationale for treatment decisions in clinical documentation	
• Team-based rounds to include consultant, resident, and nurse	
• Assignment of one consultant per patient	
• Integrate guidelines into regular training curriculum	
• Establish forum of Implementation Leaders, Champions, and any interested providers to evaluate implementation on an ongoing basis and refine as needed	
• Establish “safe space” to discuss protocol deviations and errors, including root cause analysis	

#### Step 5: Program implementation

We developed a logic model to guide the planning, implementation, and evaluation of our intervention (Table 4). To operationalize the components, we developed a project management spreadsheet, divided the responsibilities among our team, and have held biweekly videoconference calls to review progress, discuss problems, and plan next steps. ORCI team leaders have worked closely with the Tanzanian MoHCDGEC to coordinate the publication of hard copies of the guidelines and to plan a National Summit for Guideline Training. We established a linkage system, or mechanism to involve program adopters and implementers, through engendering program ownership among ORCI-based team leaders and clinical managers and training Champions.

**Table 4:** Logic model for implementation of guideline-based clinical practice at ORCI

Inputs	Outputs of activities and participants	Outcomes and impact
Staff • Project leaders (ORCI/UCSF) • Co-Investigators (ORCI/UCSF) • Implementation Champions (ORCI) • Research Coordinators (MUHAS) • Research Consultants (MUHAS/UCSF) Materials • Hard copies of guidelines • Soft copies of guidelines (AgileMD) • Publicity materials (flyers, texts) • ORCI-specific DST forms • Training materials • Hard and soft copies of questionnaires • Data collection forms for observation Experience and expertise • Training and consultation in implementation science and program evaluation • Consultation with biostatistician for questionnaire design and analysis • Experience implementing clinical protocols and DST forms at a different regional site • Existing MUHAS/ORCI/UCSF Cancer Collaboration infrastructure and experience	Distribution of materials • Hard copies to every unit and clinic room • Soft copies to every provider via smartphone application (AgileMD) • Publicity campaign with flyers and texts Education and training National Guideline Training Summit: • Raise awareness of international efforts to develop resource-stratified guidelines • Teach providers guideline content and benefits of guideline-based practice • Train providers in guideline-based practice, DST completion, documentation of rationale for treatment decisions • Train Champions to promote guideline-based practice on an ongoing basis • Integrate guidelines into existing training curricula Environmental restructuring • Champions will model and promote guideline-based practice • Integration of DST forms into clinical workflow • Assignment of one consultant per patient for greater accountability • Monthly forum to evaluate implementation and “safe space” to discuss deviations	Short-term • Increased knowledge of guidelines and skills in guideline-based practice among providers • Proficiency in completing DSTs • Shift in attitudes and beliefs toward preference for guidelines over individual experience and expertise • Increased comfort to ask peers and superiors about guideline concordance of treatment plans Medium-term • Increase in clincial decision-making based on guidelines • Routine completion of DSTs • Routine reference to guidelines in case discussions at conference • Increase in rates of guideline-concordant treatment plans made • Increase in rates of guideline-concordant treatment plans completed Long-term • Increase in cancer survival outcomes • Increase in palliative benefit and quality of life • Improved resource utilization

#### Step 6: Evaluation plan

Based on the logic model, we identified the relevant indicators of the process, outcome, and impact of our intervention. We classified and refined these indicators using the RE-AIM framework [36], orienting our evaluation toward issues relevant to program adoption, implementation, and sustainability in order to strengthen its external validity. For each indicator, we determined an appropriate research methodology for measurement among the categories of direct observation, questionnaire administration, clinical chart reviews, and cost-effectiveness analysis (Table 5). We then developed research protocols employing these methods for a selection of indicators. The first study, “aim 1,” will evaluate the activities and outputs of the implementation strategy itself in order to assess its feasibility, acceptability, fidelity versus adaptation, and sustainability using direct observation and pre-post questionnaires. The second study, “aim 2,” will evaluate the effectiveness of the implementation strategy using a pre-post design focused on guideline-concordant treatment completion, healthcare quality metrics, and survival outcomes for breast cancer and colorectal cancer. The third study, “aim 3,” will evaluate the impact of guideline-based practice on the cost of cancer treatment and resource utilization at ORCI. The results of these studies will be reported separately.

**Table 5:** Indicators of process, outcome, and impact classified using the RE-AIM framework

Reach	Proportion of ORCI providers and Champions who complete dedicated training	Direct observation
	Proportion of stakeholders who agree with the importance of guideline-based practice	Questionnaire
Effectiveness	Assessment of whether each intervention component modified its targeted barrier(s)	Mixed Methods
	Proportion of patients who complete guideline-concordant treatment	Clinical chart review
	Cure rates for curable cancers	Clinical chart review
	Survival prolongation for non-curable cancers	Clinical chart review
	Patient-reported palliative benefit and quality of life associated with treatment	Questionnaire
	Resource utilization as calculated by benefits per cost	Cost-effectiveness analysis
Adoption	Proportion of patients with a completed DST in chart	Clinical chart review
	Assignment of one consultant per patient responsible for treatment decisions	Clinical chart review
	Proportion of patients with a guideline-concordant treatment plan documented	Clinical chart review
	Rates of consultant, resident, and nurse participation in team rounds	Direct observation
Implementation	Adequacy of staff, funding, and materials to complete implementation strategy	Direct observation
Maintenance	Self-reported ability of Implementation Champions to promote guideline-based practice on an ongoing basis	Questionnaire
	Serial measurement of the “Effectiveness” and “Adoption” outcomes	Mixed Methods

## Discussion

In Tanzania and many other LMICs, shifting to guideline-based oncology practice represents a change in clinical culture and behavior, and guideline publication alone is unlikely to result in sustained adoption or measurable impact on clinical care delivery. Using the stepwise process outlined by the Intervention Mapping framework, our team successfully developed a guideline implementation strategy derived from the prominent behavior change theory COM-B/BCW. To our knowledge, this is the first report of a multifaceted theory-driven implementation strategy designed to promote the uptake of cancer treatment guidelines in sub-Saharan Africa.

Given the recent surge in resource-stratified cancer treatment guidelines internationally, there is a widespread need for dedicated guideline implementation efforts. While the intervention described here is tailored to ORCI, we surmise that there are many commonalities between the needs at ORCI and other centers in other centers in sub-Saharan Africa and in other LMICs. Indeed, the barriers to guideline implementation that we identified at ORCI are consistent with those found in a scoping review of the general literature, including lack of familiarity and agreement with guideline-based practice among physicians, insufficient access to guidelines, and constraints within clinical systems and resources [9]. Additional barriers reported in low-resource settings emerged in our formative evaluation as well, including lack of technical capacity, tradition of using expert opinion-based approaches, lack of training on guideline use, and competing priorities [8]. Moreover, the components of our intervention derived through COM-B/BCW map onto proven guideline implementation strategies, such as the *distribution of educational materials* (e.g., hard and soft copies of the guidelines) and *media* (e.g., publicity campaign) in phase 1 of our intervention, *educational meetings* and *marketing* in the form of interactive trainings at the summit in phase 2, *local opinion leaders* (e.g., Implementation Champions) and *reminders* (e.g., clinical forms) in phase 3, and *audit and feedback* in the evaluation plan [37]. These consistencies with well-established barriers and strategies enhance the validity of our process and results and predict a degree of generalizability to other settings.

Notably, however, these “proven” guideline implementation strategies have largely been tested in HICs. In a 2017 Cochrane overview of 18 systematic reviews of implementation strategies to change health worker behavior, only 1.6% of 820 primary studies took place in a low-income country, and 10% in a middle-income country [16]. While it seems plausible that guideline implementation strategies may be similarly effective in LMICs, this cannot be assumed. Our project responds to calls for “urgently needed” investigation of the transferability of evidence on implementation strategies generated in resource-rich countries, including research to learn how to best adapt strategies for LMICs as well as the discovery and evaluation of novel approaches [17].

Our rigorous evaluation plan will measure the process, outcomes, and impact of our intervention at ORCI. Importantly, we will also systematically document the modifications and adaptations made to the originally planned intervention using the Framework for Reporting Adaptations and Modifications-Expanded (FRAME) [38]. If our intervention ultimately proves effective, the next step will be to validate the approach at other sites in Tanzania and beyond, ideally using a quasi-experimental design. We hope that by providing a detailed, stepwise description of our intervention development process, others may endeavor to replicate the process in their settings.

## Data Availability

All data generated or analyzed during this study are included in this published article and a forthcoming publication reporting the results of the focus groups.

## Abbreviations

ASCO: American Society of Clinical Oncology
BCW: Behavior Change Wheel
BHGI: Breast Health Global Initiative
COM-B: Capability, Opportunity, Motivation and Behavior
D&IS: Dissemination and implementation science
DST: Diagnosis stage treatment
FRAME: Framework for Reporting Adaptations and Modifications-Expanded
HIC: High-income country
LMIC: Low- and middle-income country
MoHCDGEC: Ministry of Health, Community Development, Gender, Elderly and Children
MUHAS: Muhimbili University of Health and Allied Sciences
NCCN: National Comprehensive Cancer Network
ORCI: Ocean Road Cancer Institute
RE-AIM: Reach, Effectiveness, Adoption, Implementation, Maintenance
TIDieR: Template for Intervention Description and Replication
UCSF: University of California, San Francisco
